# Injusticia ambiental en la calidad del aire para repartidores de plataformas digitales de Bogotá, Colombia, 2021

**DOI:** 10.7705/biomedica.7162

**Published:** 2024-08-29

**Authors:** Sandra Milena Agudelo-Londoño, Luis Camilo Blanco-Becerra, Mabel Rocío Hernández, Zuly Bibiana Suárez-Morales, Laura Clemencia Mantilla-León, Nathalia Solís

**Affiliations:** 1 Instituto de Salud Pública, Pontificia Universidad Javeriana, Bogotá, D.C., Colombia Pontificia Universidad Javeriana Pontificia Universidad Javeriana Bogotá, D.C. Colombia; 2 Facultad de Ingeniería Ambiental, Universidad Santo Tomás, Bogotá, D.C., Colombia Universidad Santo Tomás Universidad Santo Tomás Bogotá, D.C. Colombia; 3 Escuela de Ciencias Humanas, Universidad del Rosario, Bogotá, D.C., Colombia Universidad del Rosario Universidad del Rosario Bogotá, D.C. Colombia

**Keywords:** condiciones de trabajo, calidad del aire, equidad en salud, justicia ambiental, justicia social, tecnología de bajo costo, material particulado, Working conditions, air pollution, health equity, environmental justice, social justice, low-cost technology, particulate matter

## Abstract

**Introducción.:**

La calidad del aire es un asunto de interés para la salud pública por su rápido deterioro en los países de bajos y medianos ingresos, y los efectos del aire contaminado en la salud de las poblaciones.

**Objetivo.:**

Explorar las condiciones de la calidad del aire en las que los repartidores de plataformas digitales desarrollaron su trabajo en las localidades de Kennedy y Usaquén de Bogotá durante el 2021.

**Materiales y métodos.:**

Se llevó a cabo un estudio mixto, paralelo y convergente, basado en cuatro fuentes de información: 1) observación etnográfica en cinco ubicaciones comerciales de las dos localidades; 2) monitoreo de PM_10_ y PM_2.5_ en 56 rutas de reparto, empleando un equipo de bajo costo; 3) bitácoras diarias de los recorridos que apoyaron la interpretación de los datos del equipo, y 4) entrevista semiestructurada con el rutero para explorar sus percepciones frente a los peligros durante los recorridos.

**Resultados.:**

Se identificaron diferencias en las condiciones de trabajo, las percepciones y las exposiciones a material particulado de los repartidores entre las dos localidades de estudio que constituyeron fuentes de injusticia ambiental. Los recorridos que realizaron los repartidores en la localidad de Kennedy registraron mayores concentraciones de PM_10_ y PM_2.5_. Las fuentes de contaminación atmosférica identificadas por los repartidores mostraron los peores parámetros en Kennedy.

**Conclusiones.:**

Se evidenció que la calidad del aire, el equipamiento urbano, la infraestructura vial, las fuentes móviles y la ubicación geoespacial son elementos que marcan la presencia de injusticia ambiental para los repartidores. Para disminuir esta inequidad, es necesario que las plataformas de reparto digital y el gobierno distrital implementen estrategias que reduzcan la exposición y la emisión de contaminantes del aire con el fin de proteger la salud de los repartidores de plataformas.

## Abstract (en)

**Introduction.:**

Air quality is a matter of interest for public health due to its rapid deterioration in low- and middle-income countries and the effects of polluted air on the health of populations.

**Objective.:**

To explore the air quality conditions in which digital platform delivery workers carry out their work, evaluating the localities of Kennedy and Usaquén in Bogotá, 2021.

**Materials and methods.:**

We developed a mixed parallel convergent study based on four sources of information: 1) Ethnographic observation in five commercial locations of the two localities; 2) Monitoring of PM_10_ and PM_2.5_ in 56 delivery routes using a low-cost sensor; 3) Daily logs of the routes to support the device data interpretation, and 4) A semi-structured interview applied to the drivers to explore their danger perception during the routes.

**Results.:**

We identified elements causing environmental injustice among digital platform delivery workers between the two study locations. The routes made by the delivery drivers in the locality of Kennedy registered higher concentrations of PM_10_ and PM_2.5_, compared to the values observed in Usaquén. The sources of air pollution identified by the delivery drivers through ethnographic observation and the router logbook showed the worst parameters in Kennedy.

**Conclusions.:**

We evidenced that air quality, urban equipment, road infrastructure, mobile sources, and geospatial location are elements that mark the presence of environmental injustice for the digital platform delivery drivers in the studied localities. To reduce this inequity, it is necessary for digital delivery platforms and the district government to implement strategies that reduce the exposure and emission of air pollutants to protect the health of digital platform delivery workers.

La calidad del aire es un asunto de interés para la salud pública a nivel mundial ^1^, en especial, por su rápido deterioro en los países de bajos y medianos ingresos ^2^ y sus efectos en la salud de las poblaciones que se expresan en una mayor carga de morbilidad y mortalidad, sobre todo en los grupos más vulnerables, como adultos mayores e infantes ^2^. De ahí que sea central la pregunta del efecto diferencial de la calidad del aire sobre la salud de las personas, por ejemplo, sobre aquellos que se desempeñan como repartidores de plataformas digitales ^3^.

En grandes ciudades del sur global, como Bogotá -con 7,9 millones de habitantes en el 2022 ^4^-, la contaminación del aire por partículas menores de 10 μm (PM_10_) ha aumentado levemente en los últimos años ^5^, además de exceder el umbral anual (15 μg/m^3^) según la “Guía de calidad del aire”, establecida por la Organización Mundial de la Salud (OMS). Esto ha significado una mayor exposición de las poblaciones a posibles efectos nocivos sobre su salud ^6^^-^^8^. Actualmente, la evidencia sobre la exposición a contaminantes en el aire y sus impactos sobre la salud de los repartidores de plataformas digitales es escasa.

En la ciudad, el reparto de domicilios mediado por plataformas digitales es un fenómeno en crecimiento ^9^, ejercido principalmente por jóvenes, mujeres, migrantes y personas que no encuentran empleos formales, en medio de economías cada vez más informales ^10^, por lo que la salud de estos repartidores debería ser un asunto de interés de los gobiernos y la sociedad en general.

El trabajo de reparto mediado por plataformas digitales promete diversas ventajas a los repartidores, entre otras, la flexibilidad de las jornadas, la autonomía del repartidor y los ingresos inmediatos. Sin embargo, se ha evidenciado que el control algorítmico de las plataformas de reparto, la ausencia de legislación laboral y otras circunstancias propias del modelo de negocio digital ^11^ precarizan las condiciones reales de trabajo de los repartidores exponiéndolos diferencialmente a riesgos específicos de su oficio.

En ese sentido, un riesgo particular del reparto mediado por plataformas digitales es que requiere ser ejecutado físicamente en la calle ^12^, con los peligros propios de los espacios urbanos, como la posibilidad de incidentes viales, las condiciones climáticas (lluvia y radiación solar), la inseguridad por robos y la contaminación del aire.

La calidad del aire se mide en función de las concentraciones de contaminantes como el material particulado, además de los olores ofensivos y el ruido que, en el caso de las ciudades, se debe a las edificaciones, el equipamiento urbano, la infraestructura vial, las fuentes móviles y la ubicación geoespacial ^13^. De esta manera, las poblaciones están expuestas socio-espacialmente ^14^ a distintas calidades de aire ^15^. No obstante, estas condiciones no son solo naturales -como es el caso de la ubicación geográfica o las corrientes de aire según la topografía de la región-, sino también, producto de políticas públicas, acciones y omisiones gubernamentales que expresan lógicas desiguales e, incluso, inequitativas hacia las poblaciones.

El estudio de lo ambiental desde una perspectiva de equidad no es nuevo. La inequidad ambiental aborda “todo perjuicio indebido en la distribución de las cargas ambientales entre los grupos de población” que tienen el potencial de afectar o promover la salud ^16^, y reflejan un juicio moral implícito según el cual las desigualdades ambientales son injustas, “evitables, inmerecidas y remediables” ^17^ y necesitan ser rectificadas como un imperativo moral ^14^.

El concepto de justicia ambiental, que se nutre de diversas vertientes teóricas -desde los debates sobre justicia distributiva de Rawls en relación con los bienes y riesgos ambientales hasta las perspectivas tradicionales de la epidemiología ambiental ^18^-, se preocupa por “la distribución equitativa de las cargas y beneficios ambientales entre todas las personas de la sociedad considerando el reconocimiento de la situación comunitaria, las capacidades de las personas y su participación en la adopción de las decisiones que los afectan” ^19^. Así, una apuesta por la justicia ambiental para los trabajadores de plataformas orienta este análisis sobre las condiciones diferenciales de la calidad del aire en los lugares donde desempeñan su actividad mediante la comparación de dos localidades geográfica, económica y socio-ambientalmente opuestas de Bogotá: Kennedy y Usaquén.

Bogotá, la capital de Colombia, es una ciudad sobre la cordillera de los Andes a 2.600 msnm, organizada socio-espacialmente en 20 localidades. Según la “Red de monitoreo de la calidad del aire de Bogotá” (RMCAB), las localidades de Kennedy (al suroccidente de la ciudad) y Usaquén (al nororiente) presentan históricamente los valores promedio más distantes de material particulado: de 10 (PM_10_) y de 2,5 μm (PM_2.5_); en el caso de Kennedy, supera el valor guía anual de la OMS ^2^. Además, estas dos localidades son muy distintas en términos socioeconómicos y demográficos. Kennedy es la localidad con mayor población de la ciudad, cuenta con más de un millón de habitantes, de los cuales más del 90% son de ingresos bajos a medios ^20^ y presenta una gran ocupación de trabajadores informales. Usaquén, por su parte, tiene 500.000 habitantes donde solo 17.017 hogares (6,4 %) se consideran pobres y una gran tasa de ocupación formal ^21^. Sumado a esto, las dos localidades muestran una gran concentración de repartidores por la presencia de grandes superficies comerciales.

En ese sentido, el análisis de la injusticia ambiental propuesto indaga por el rol de los lugares donde se desarrolla el trabajo, en la posible distribución inequitativa de las cargas ambientales sobre grupos poblacionales específicos. Se reconoce así que todo trabajo en la calle implica diferentes exposiciones ambientales, en comparación con otros trabajos y ocupaciones desarrollados bajo techo.

Sin embargo, ya que ser repartidor es un trabajo móvil con gran demanda respiratoria, requiere ser analizado en sí mismo, en especial, porque los repartidores deben hacer sus entregas contra reloj en incesantes desplazamientos (la mayoría en bicicleta) en las mismas zonas. Los lugares donde realizan sus actividades de reparto no son seleccionados por decisión propia del repartidor, sino que hacen parte de complejas redes de distribución de productos y servicios, de dinámicas de asignación geográfico-algorítmicas invisibles para el repartidor y controladas por las plataformas digitales de reparto.

Teniendo en cuenta estas particularidades, el objetivo del presente trabajo fue explorar, en clave de justicia ambiental, las condiciones de la calidad del aire bajo las cuales los repartidores de plataformas digitales desarrollan su trabajo en Bogotá, comparando dos localidades socio-económicamente opuestas en 2021.

## Materiales y métodos

Se realizó un estudio mixto, paralelo y convergente ^22^, para explorar las condiciones de la calidad del aire entre los lugares con las mejores - Usaquén- y las peores -Kennedy- concentraciones promedio de material particulado (PM_2.5_ y PM_10_) en Bogotá, en el 2019, según los datos de la red de monitoreo de la calidad del aire de Bogotá RMCAB.

El estudio se basó en cuatro fuentes de información. La primera, que abarcó del 23 de julio al 12 de agosto de 2021, consistió en un ejercicio de observación etnográfica en cinco lugares de vocación comercial reconocidos por su gran concentración de repartidores. Con ellos, se conversó sobre sus percepciones frente al ambiente y a los contaminantes a los cuales se exponían; estas interacciones constituyeron parte del reporte etnográfico.

Los siguientes fueron los datos obtenidos de la segunda fuente de información. Con los repartidores seleccionados y por muestreo por conveniencia, en total, se reconstruyeron 56 rutas de reparto en las localidades seleccionadas (27 en Usaquén y 29 en Kennedy). En estas rutas, se midieron los valores de PM_10_ y PM_2.5_ con el equipo de monitoreo de material particulado de bajo costo AirBeam® ^23^, ubicado a la altura del pecho de un exrepartidor (en adelante, rutero) quien recorrió las rutas en bicicleta. La medición se hizo en Usaquén del 6 al 9 agosto del 2021 y, en Kennedy, del 10 al 15 de agosto del 2021, entre las 11:00 y las 19:00 horas, periodo en el que es mayor la demanda de pedidos.

La tercera fuente de información fueron las bitácoras diarias de las rutas recorridas por el rutero para obtener información contextual que apoyara la interpretación de los datos del equipo de medición.

Finalmente, se llevó a cabo una entrevista semiestructurada con el rutero, para explorar sus percepciones de los peligros a los que está expuesto durante la ejecución de las rutas. El presente estudio se desarrolló durante el tercer trimestre del año 2021, cuando aún continuaba la emergencia sanitaria por la COVID-19, prorrogada hasta el 31 de agosto de 2021 mediante la Resolución 738 de 2021, emitida por el Ministerio de Salud y Protección Social. A fecha del 1° de agosto del 2021, Bogotá alcanzaba la cifra de cinco millones (cerca del 50 %) de vacunas aplicadas contra el SARS-CoV-2 entre sus habitantes para obtener la inmunidad de rebaño ^24^; además, presentaba una reactivación económica significativa y aumento del empleo, con un crecimiento de la economía del 10,3 % y una recuperación del empleo cercana al 92 % ^25^.

Los datos de las mediciones de PM_10_ y PM_2.5_ fueron almacenados en Microsoft Excel 2013. Se verificaron la completitud, la coherencia interna y la calidad de los datos. Las bases depuradas se analizaron en Stata™, versión 11 y SPSS™, versión 28. Para estimar las diferencias en las concentraciones de PM_10_ y PM_2.5_, medidas por segundo entre las dos localidades, se aplicó la prueba no paramétrica U de Mann-Whitney con un nivel de significancia del 5 %. Los análisis geoespaciales de las rutas en las dos localidades se graficaron con el programa ArcGis™, versión 10.8.

El audio de la entrevista se transcribió mediante Verbatim, y las observaciones etnográficas y las bitácoras, en Microsoft Word. Los datos se codificaron manualmente mediante análisis de contenido inductivo, buscando extraer conclusiones sustanciales. Todos los informantes participaron voluntariamente después de otorgar su consentimiento informado. Los datos personales fueron anonimizados desde su obtención. La triangulación metodológica de las fuentes se logró por medio del diálogo entre los análisis cuantitativos y las fuentes interpretativas obtenidas ^26^. Se logró la saturación teórica. El trabajo contó con la aprobación del Comité de Ética e Investigación de la Universidad del Rosario .

## Resultados

Para explorar las condiciones de la calidad del aire a las que se exponen los repartidores de plataformas digitales en Bogotá, en términos de justicia ambiental, se presentan los análisis triangulados del monitoreo del material particulado (PM_10_ y PM_2.5_) en las dos localidades seleccionadas, junto con las observaciones etnográficas, las bitácoras y la entrevista desarrollada con el rutero en 2021.

El muestreo total del material particulado, con el equipo AirBeam®, fue de 27.550 segundos en Usaquén y de 26.638 segundos en Kennedy. El promedio de concentración por segundo fue de 9,67 μg/m^3^ en Usaquén y de 14,32 μg/m^3^ en Kennedy. En Usaquén, en 14 minutos (equivalentes al 3 % de los segundos), se encontraron picos de concentraciones por encima de los 45 μg/m^3^,y en Kennedy, en 22 minutos (equivalentes a 5 % de los segundos), se superó el valor estándar establecido por la OMS ^2^.

Con respecto al PM_2.5_, en Usaquén se midieron 27.050 segundos y, en Kennedy, 26.638 segundos. El promedio de concentración por segundo fue mayor en Kennedy (9,94 μg/m^3^) que en Usaquén (6,78 μg/m^3^). En Kennedy, en 89 minutos se presentaron picos mayores que el valor de 24 horas de 15 μg/m^3^ establecido por la OMS ^2^, mientras que en Usaquén este valor se superó en 45 minutos.

**Figura 1:**
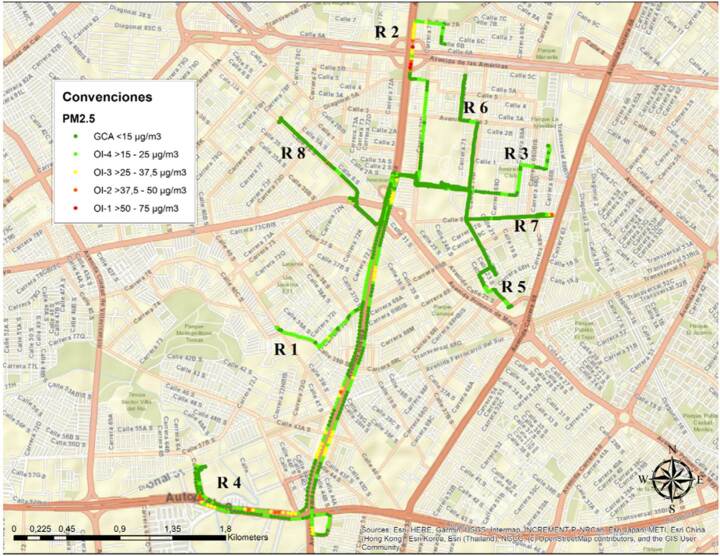
Concentraciones por segundo de material particulado fino (PM_2.5_) registradas en cada uno de los recorridos realizados el tercer día en la localidad de Kennedy, 2021

Las diferencias entre las concentraciones de PM_10_ y PM_2.5_ en las dos localidades fueron significativas (p < 0,000), con mayores picos de exposición en Kennedy. Como el AirBeam® facilita la identificación espacial de los puntos de mayor concentración de contaminantes, en la figura 1 se presentan los valores por segundo de PM_2.5_ del tercer día de observación, cuando se registraron los datos más altos. Ese día se ejecutaron ocho rutas en Kennedy, con un acumulado de 56 minutos de exposición a PM_2.5_ en concentraciones mayores de 15 μg/m^3^ y una concentración máxima por segundo de 273 μg/m^3^.

Para contextualizar el análisis de las concentraciones de material particulado desde las percepciones construidas en los sitios, se identificaron las principales fuentes emisoras en ambas localidades mediante observaciones etnográficas y la bitácora del rutero. Así, la reparación de la malla vial, las vías no pavimentadas, las zonas de construcción, la poda de zonas verdes, las emisiones de los buses y otros vehículos pesados, y los puestos de comidas rápidas en la calle que utilizan carbón o madera, fueron identificados en las diferentes fuentes de información, reportándose peores condiciones para Kennedy. En ese sentido, en el cuadro 1 se muestran los extractos del diario de observación etnográfica, las bitácoras de ruta y la entrevista con el rutero, organizados según los principales contaminantes del aire percibidos durante el trabajo de reparto y la observación en sitio.

**Cuadro 1:** Percepciones sobre los contaminantes del aire en el trabajo de reparto digital en dos localidades de Bogotá, Kennedy y Usaquén, 2021

Contaminante identificado del aire	Kennedy	Usaquén
Material particulado (gases, polvo, humos)	“La irritación de los ojos lo noté en Kennedy, por los lentes, por las gafas, de pronto no me afecta tanto, pero igual si se notaba el polvo al final del día” (Entrevista rutero, 2021). “Por lo menos en los aspectos de la piel, al final del día, por el sudor y las partículas de polvo llegó a la casa más sucio” (Entrevista rutero, 2021). “Cuando los vehículos particulares y de transporte público pasan, se levanta una gran cantidad de material particulado, incluso hay días que se puede observar el polvo acumulado sobre las hojas de los árboles. Cerca a esa vía queda un parqueadero que es un lote sin pavimento donde se guardan buses. Me dicen que en la autopista sur se levanta bastante polvo, por el tráfico pesado y los lotes destapados que están a un lado de la vía”. (Observación etnográfica, agosto 10, 2021). “Ambiente con nube de polvo por tráfico, obras en la vía y fumadores durante las rutas [de reparto]” (Bitácora rutero, agosto 15 de 2021).	“No es igual hacer entregas en la mañana o en la tarde: el aire se siente mucho más limpio por la mañana” (Entrevista rutero, 2021). “Ambiente con polvo por tráfico, construcciones, venta de arepas y fumadores durante las rutas” (Bitácora observación, agosto 9 de 2021). “Aunque esta es un área con alto flujo vehicular, no sentí el aire tan contaminado mientras estuve en la plazoleta del Carulla o en los parques aledaños”. (Observación etnográfica, 23-24 de julio de 2021).
Ruido	“Las rutas [de reparto] tenían [atravesaban] áreas residenciales, algunas pasaban por avenidas con tráfico alto todo el tiempo o tenían construcción para el metro, entonces las máquinas generaban ruido permanente” (Entrevista rutero, 2021).	“Los sonidos más frecuentes son de los automóviles y motos constantes. Cerca hay unos locales de tipo bar, donde en la noche aumenta el ruido”. (Observación etnográfica, julio 23-25, 2021).
Olores ofensivos	“Afirman [los repartidores] que se siente la contaminación... por la cercanía del matadero. También mencionan que, en cierta época del año, es fuerte el olor que emana del río Tunjuelo. (Observación etnográfica 26 de julio, 2021)	

Fuentes: observación etnográfica, bitácora y entrevista del rutero

Con base en lo reportado en el cuadro 1, fue posible identificar las percepciones sobre: a) los contaminantes, b) las fuentes emisoras, c) las condiciones de exposición percibidas, y d) las molestias referidas durante el trabajo de reparto, como insumos para ampliar la lectura de la calidad del aire en clave de justicia ambiental en las localidades de estudio.

Los vehículos se destacaron como la mayor fuente percibida de contaminación auditiva en ambas localidades, especialmente en las horas de mucho tráfico. El rutero identificó, además, a los bares en Usaquén y a la maquinaria pesada en Kennedy como fuentes de ruido relevantes. Por su parte, los olores ofensivos, se reportaron solo en Kennedy, por su cercanía con un frigorífico (área de sacrificio de bovinos y porcinos) y el río Tunjuelo. En relación con las molestias percibidas, se encontró que la irritación de los ojos y la suciedad del cuerpo al final de la jornada fueron incomodidades comunes asociadas con el material particulado presente en las vías por donde transitan. Si bien estas molestias se reportaron en las dos localidades, fueron más frecuentes en Kennedy (cuadro 1). Vale la pena señalar que los contaminantes identificados, sus fuentes y las molestias percibidas en las rutas de reparto, fueron similares entre las diferentes fuentes de información cualitativa.

En consecuencia, la calidad del aire percibida en las rutas estudiadas de reparto digital generó molestias durante las observaciones y esta percepción fue diferencial entre las localidades, siendo peor para Kennedy. Lo anterior fue coherente con el monitoreo de contaminación medido con el equipo de bajo costo AirBeam®.

## Discusión

Con este trabajo se identificaron diferencias significativas en las concentraciones de material particulado medidas en las dos localidades, con peores parámetros de calidad del aire para la localidad de Kennedy. En el mismo sentido, los repartidores percibieron diferencialmente que la contaminación del aire de las localidades dependía del parque automotor rodante, del estado de la malla vial, de las edificaciones y del equipamiento urbano, así como de la cercanía con fuentes hídricas contaminadas y lugares de sacrificio animal -considerando las diferencias socioeconómicas y geográficas de las localidades- que evidencian las condiciones de trabajo ambientales desiguales para los repartidores de plataformas digitales entre las dos localidades y las lógicas de injusticia ambiental en el reparto digital ^19^.

Esta relación entre calidad del aire, equipamiento urbano y condiciones socioeconómicas (considerando que Kennedy es una localidad con menores ingresos que Usaquén), es congruente con una revisión bibliográfica en la cual se encontraron mayores concentraciones de contaminantes del aire en los lugares donde habitan las comunidades de más bajo nivel socioeconómico ^27^. En India, uno de los países más contaminados del mundo, se encontraron mayores concentraciones de PM_2.5_ en los distritos donde habitaban las castas y tribus registradas como las más pobres del país y con la mayoría de niños pequeños ^28^. En un estudio desarrollado en la región metropolitana de Santiago de Chile, también se identificó que, en las zonas con peor calidad del aire, residían las personas de más bajos niveles socioeconómicos y que el aire contaminado afectaba inequitativamente a la población ^29^.

En un estudio realizado en 2013, en las cuatro localidades con mayores concentraciones de material particulado en Bogotá, se encontró que más del 80 % de las personas tenía una percepción negativa sobre la calidad del aire y más de la mitad señaló al transporte como la principal fuente de contaminación ^30^. En el mismo sentido, un trabajo realizado con vendedores informales del centro de Medellín en el 2020, permitió identificar a los vehículos, las chimeneas y la quema de basura o llantas, como fuentes que aumentaban las emisiones, en especial, en las horas de la tarde y en zonas de mucho tráfico, como paraderos de buses y vías principales ^31^.

A pesar de las diferencias metodológicas entre los estudios mencionados y las condiciones de las dos ciudades (Medellín y Bogotá), los hallazgos anteriormente descritos fueron similares a los resultados de este trabajo respecto a la percepción de los ciudadanos frente a la calidad del aire, quienes no se limitan a considerarla buena o mala, sino que también implican las posibles fuentes de emisión y las molestias percibidas, es decir, los ciudadanos no solo son receptores, sino gestores del aire que respiran ^32^. Por esto, en las investigaciones sobre los repartidores de plataformas digitales es necesario vincular otros actores al debate, otras lógicas en las mediciones y en las percepciones y otras preguntas, que permitan avanzar en un modelo de gobernanza del aire en clave de justicia ambiental.

Cabe anotar que, para medir la calidad del aire se requieren equipos especiales que pueden ser costosos. Por esto, el uso de equipos de bajo costo, como el utilizado en esta investigación, ha sido una preocupación en los últimos años. Prueba de ello son los trabajos que se han venido desarrollando en el campo de la ciencia ciudadana, como el de espacios libres de humo de tabaco de Kentucky ^33^ y el ejecutado en vecindarios de bajos recursos económicos con adultos mayores ^34^, así como en ciclorrutas en Medellín (Colombia) ^35^. Si bien equipos como el Airbeam® no tienen la misma precisión y exactitud que aquellos de los sistemas de vigilancia de la calidad del aire, sí suministran una aproximación de la exposición real de las personas en sus lugares de permanencia y permiten obtener buenas mediciones cuando son usados según el entorno ^35^^-^^37^. Por esta razón, son una herramienta práctica y económica para estimar las concentraciones de contaminantes en el aire en espacios interiores y exteriores.

Como resultado del estudio, surgen recomendaciones sobre diferentes aspectos. Desde una lógica próxima a la de los repartidores, una de ellas está encaminada a mejorar sus condiciones de protección personal, minimizando la exposición al material particulado en límites no seguros para crear, así condiciones de menor desventaja en la localidad de Kennedy que en la de Usaquén; a las plataformas de reparto digital se les recomienda reducir la exposición a contaminantes del aire de los repartidores mediante la formulación de rutas seguras, en donde las concentraciones de material particulado sean las de menor valor ^36^^,^^38^^,^^39^, además de entregar elementos de protección personal dirigidos a mitigar su exposición.

Las condiciones de trabajo y empleo de los repartidores, que constituyen una desigualdad injusta y evitable entre las dos localidades, podrían exponerlos a mayores vulnerabilidades en contextos que ya son ambientalmente riesgosos para la salud, como el demostrado con las mediciones de la calidad del aire en la localidad de Kennedy. Concentrarse en la respuesta ocupacional deja la desigualdad estructural sin resolver, por lo que una de las recomendaciones sería poner en marcha el “Plan estratégico para la gestión integral de la calidad del aire de Bogotá” (Plan Aire 2030), por parte del Distrito y con énfasis en Kennedy, como una hoja de ruta para la incorporación de tecnologías de cero y bajas emisiones a la flota vehicular del transporte público, la reducción de emisiones de transporte urbano de carga, la pavimentación de vías, la priorización ambiental, la mejora del barrido mecánico en la malla vial y el aumento de vegetación en la localidad y la ciudad. Con ello, se podría mejorar la calidad del aire al que están expuestos los repartidores de plataformas digitales de Bogotá.

Finalmente, se requiere la reflexión y una gestión motivada por parte de los tomadores de decisiones locales, que incluyan a los repartidores de plataformas, a los demás trabajadores en la calle y, en general, a la población de la localidad, para adecuar su localidad como un espacio urbano saludable. Esta sería una apuesta por la justicia ambiental de los trabajadores de plataformas.
